# Work Ability and Work Participation Statuses of the Working-Age Population in Finland: A Register-Based Clustering Study

**DOI:** 10.1007/s10926-025-10303-5

**Published:** 2025-06-06

**Authors:** Vaula Siltala, Mikko Henriksson, Matti Joensuu, Jenni Ervasti, Elina Ahola, Jarno Turunen

**Affiliations:** Finnish Institute of Occupational Health, Helsinki, Finland

**Keywords:** Work ability, Work disability, Employment, Unemployment, Cluster analysis

## Abstract

**Purpose:**

Work ability is a complex concept without a single golden standard measure. This study explores the possibility to use register data to form distinct work ability clusters and examine the levels of work participation between these clusters, while considering the co-occurrence of factors contributing to work ability within the population.

**Methods:**

We used register data from a 90% sample of the Finnish working-age population in 2021 (ages 20–64, *n* = 2 920 099) to operationalize work ability based on literature. Employing K-Prototypes clustering, we identified distinct clusters of work ability. In this cross-sectional study, we then examined work participation by assessing the number of months spent in employment, unemployment, and receiving disability benefits within each cluster.

**Results:**

The analysis resulted in 11 distinct clusters. The clusters differed from each other in age, sex, educational attainment, occupation, household size and family type, and health. The average number of months spent in different work participation statuses in 2021 varied across these clusters with employment ranging from 7.3 to 11.6 months, unemployment from 0.3 to 2 months, and disability benefits from 0.2 to 2 months per year.

**Conclusions:**

Operationalization of the multifaceted concept of work ability using register data resulted in the identification of different clusters within the Finnish working-age population. Differences in various work ability-related variables highlight the complexity of work ability. Notably, the variation in work participation statuses among these clusters underscores the nuanced ways in which these factors interact to influence employment, unemployment, and reliance on disability benefits.

## Introduction

OECD countries are experiencing rapid aging, potentially hindering living standards and leading to unsustainable increases in social and health expenditures [1]. Alongside aging populations, the rising prevalence of mental health disorders [2] and insufficient responses to musculoskeletal problems [3, 4] pose significant challenges to work ability and work participation of working-age and aging population, presenting complex issues for decision-makers worldwide. The shift towards more knowledge-based work [5], the intensification of work [6], and increasing competency requirements place greater demands on employees. Global labor force participation has declined by nearly 1% over the last decade, with low-income and upper-middle-income countries experiencing the largest decreases, while lower-middle-income and high-income countries have seen the largest increases [7]. Meanwhile, participation rates among individuals aged 55–64 years and those 65 and older have risen worldwide, and especially in high-income countries such as Finland [7]. The increasing challenges to the work ability of the working-age population, pressure to increase retirement ages [1], along with projected labor shortages [8, 9], highlight the need for a deeper exploration of the relationship between work ability and work participation to support work participation for groups such as older individuals, those with work disabilities, caregivers, and individuals with chronic health conditions.

A universally accepted definition of work ability is lacking in scientific literature. In their review, Lederer et al. [10] propose a work ability concept map, based on published definitions of work ability. It extends beyond the traditional focus on work disability, providing more comprehensive understanding on the concept of work ability and aiding researchers in selecting complementary indicators for it. The indicators previously used and discussed by Lederer et al. [10] primarily focus on measures of work disability. They advocate for incorporating metrics such as productivity at work and well-being. Furthermore, they propose using combined indicators or indexes that capture different dimensions from the concept map. Cadiz et al. [11] synthesize the existing cross-disciplinary literature and emphasize the need for future advancement in research on work ability. They advise against using single measures, as these cannot capture the complex, multidimensional nature of work ability, and emphasize the need to clearly establish multiple factors contributing to work ability and its outcomes. Together, these insights call for a shift in focus from work disability to work ability, emphasizing capabilities over limitations. Moreover, they emphasize the importance of capturing the complex phenomenon of work ability, including its dynamic interactions and the potential co-occurrence of contributing factors within the population. This shift in perspective could equip policymakers with valuable tools to enhance work participation and address labor market needs more effectively.

We present an exploratory national register-based cross-sectional study to expand the analysis of work ability by adopting a perspective that considers the co-occurrence of different work ability factors, rather than focusing on individual factors such as age or education, which have been common in the related literature on working life expectancy (see, e.g., Pedersen & Bjorner [12]; Burdorf et al. [13]; Solovieva et al. [14]). Building on the concept map by Lederer et al. [10], who offer a comprehensive framework for understanding work ability by categorizing it into levels and dimensions, we operationalized work ability using cross-sectional administrative register data of the working-age population in Finland. As presented by Lederer et al. [10], the framework includes not only determinants of work ability but also inhibitors and facilitators of the process of moving out of work disability, as well as outcomes resulting from work ability or disability. The levels they identify are the individual, organizational, and societal levels. At the individual level, dimensions encompass physical, mental, social, demographic, educational, cultural/symbolic, and financial aspects. At the organizational level, the mesosystems include “work,” “insurance,” “healthcare,” and “community.” Finally, at the societal level, they consider “macro infrastructures, systems, and societal dynamics.” As the data from administrative registers are on individual level, our focus in this study is also on the individual level, where we operationalize the aforementioned dimensions using register data from the Finnish working-age population.

In this cross-sectional descriptive study, we applied the framework developed by Lederer et al. [10] to provide a comprehensive description of working-age people according to factors related to work ability. We operationalized individual-level factors and determinants using register-based proxies and then used cluster analysis to group individuals. These proxies reflect factors and determinants related to work ability. After forming the clusters, we interpreted them and analyzed the variance of work participation in clusters, specifically examining the number of annual months of employment, unemployment, and time spent on disability benefits. This approach, based on cross-sectional register data from 2021, aimed to describe and understand the collective contribution of work ability-related factors and determinants on individuals, the dynamic interactions of these factors, and how individuals group into clusters, along with their relationships to variations in work participation. As such, our approach addresses the limitations of a narrow focus by incorporating various dimensions beyond the determinants of work ability. The final product of our study will illustrate a more capability-focused view of work ability and demonstrate how clustered data can be utilized by various stakeholders and policymakers. As a result, the entire population of working-age individuals can be classified into various work ability clusters, and thus the concept of work ability is not limited to merely identifying limitations and shortcomings. This approach offers a preventive method to examine potential declines in work ability, as the scope of work ability is broader than that of traditional examinations using work disability-related measures.

## Materials and Methods

### Data Source and Sample

We used *The state of work ability in Finland* data [15] which is a 90% (random) population sample of the Finnish working-age population, to operationalize the concept of work ability. At the time of sampling, total population sampling was not available due to data security reasons. For this study, we examined people aged 20–64 at the end of 2021. The register-linkage data for 2021 included 2,920,099 people and consists of the register data of Statistics Finland, the Social Insurance Institution of Finland, the Finnish Centre for Pensions, and the Finnish Institute for Health and Welfare.

### Variables Used

#### Operationalization of Work Ability Using Proxies from Register Data

Based on the concept map by Lederer et al. [10], the register-based proxies for factors and determinants related to work ability in different dimensions included firstly demographic proxies age, sex, native language, and type of municipality of residence. Proxies related to educational/vocational dimension included: the highest level of education obtained, occupational group based on classification of occupations. Social dimensions proxies were household size, family type, and number of children under 18 in the family. For all proxies, see Table 1. Variables for the proxies were derived from the register of Statistics Finland. The selection of the above-mentioned proxies was fairly straightforward, as there were not many options in the data. However, the selection of health variables was more complicated.

**Table 1:** Register-based proxies for factors and determinants related to work ability according to the specified dimensions by Lederer et al. [10]

Dimension	Determinant of work ability	Register-based proxy with values
Demographic		
	Sex	Male, Female
	Age	Number of years
	Native language	Finnish, Swedish, other
	Type of municipality of residence	City, densely populated, rural area
Educational, Vocational		
	Level of education	Primary or missing information, secondary, higher degree
	Classification of occupations	10 different occupational groups and a “missing information” group
Social		
	Household size	1, 2, 3, or more persons
	Family type	Living alone, a couple, a couple with children, a single parent
	Children aged under 18	0, 1, 2–3, 4, or more
Physical/mental, behavioral, emotional		
	Number of health challenges	0, 1, or 2

Adequate health is one of the main factors necessary for being able to work. However, a vast number of people participate in the workforce despite experiencing some form of health decline, whether it be a diagnosed condition or a transient health issue. There are various health-related variables in the registers, most of which are not directly related to or indicative of work ability. We selected the following two variables to represent physical and mental dimensions and thus, health challenges: the purchase of medications under the special reimbursement system of the Social Insurance Institution [16] and the use of specialized health care [17]. Among the various health-related variables, we interpret that there are grounds to claim that, at the population level, these could be possible indicators of health challenges that are also indications of a decline in work ability. These variables suggest health challenges that require either specialized healthcare or specialized medication.

We constructed the health challenges status to comprise three levels based on the number of health challenges identified by the selected variables: (1) No health challenges, (2) One health challenge (at least one purchase of medications under Special Reimbursement OR the use of specialized health care at least once a year), and (3) Two health challenges (at least one purchase of medications under Special Reimbursement AND the use of specialized health care at least once a year). This health challenges status offers a multidimensional interpretation of health for the indicator. By employing these criteria, we focus on aspects potentially related to work ability, recognizing that not all health-related information may pertain directly to work ability.

We also conducted a sensitivity analysis that excluded the measure for health challenges from register-based proxies and determinants related to work ability.

#### Outcome Variables

Three distinct work participation statuses from the year 2021 were used as outcome variables. First, work participation was measured by calculating the number of months employed. Second, unemployment was measured through the months spent unemployed. Third, the use of disability benefits was measured by the number of months receiving such benefits, which included days on full- or part-time sickness allowance and disability pension [18]. It should be noted that the work participation statuses employment, unemployment, and the use of disability benefits are not mutually exclusive. An individual can simultaneously be employed (or unemployed) and receive, for example, sickness allowance. Additionally, these categories do not encompass all possible benefits or statuses in Finland, such as other types of benefits or being a student. All outcome variables were derived from administrative register data on days spent in various statuses: employed, unemployed, or receiving disability benefits.

### Data Analysis

We began by describing how each work ability-related proxy used in the clustering analysis individually relates to work participation statuses at the population level. Next, we assessed the work ability of the working-age population by operationalizing factors and determinants identified as related to work ability [10] from the register data with a particular focus on individual-level dimensions. Specifically, we clustered individuals in the data based on these work ability-related proxies using K-prototypes clustering method [19]. This method allowed us to assign each observation to a single cluster by incorporating both continuous and categorical variables. In K-prototypes clustering, we used Euclidean dissimilarity measure for the continuous variable (age) and simple matching for the categorical variables (all other variables). Initialization in clustering was made according to Huang [19, 20].

We determined the optimal number of clusters by computing the clustering cost, defined as the sum of distances of all points to their respective cluster centroids, aiming to minimize the sum of distances. See the Appendix for further information on cluster solutions and associated distance measures. We conducted clustering analyses for solutions ranging from 2 to 20 clusters using the entire dataset and identified the optimal number of clusters by pinpointing the local minimum value of the sum of distances. These local minima were qualitatively assessed, and the first local minimum served as our primary criterion for clustering solution [19].

Following the identification of clusters, the work participation statuses were measured for each of them. Specifically, we calculated the average duration, measured in months, spent in various statuses (employed, unemployed, on a disability-related benefit) for each cluster. Clustering was performed on Python version 3.9.13 using package kmodes (0.12.2). All other analyses were performed on Stata 18.0.

## Results

### Work Participation Statuses per Register-based Proxies for Work Ability

Table 2 reports the annual employment months, unemployment months, and months spent in disability benefits at the population level for each work ability-related register proxy used in the clustering analysis.

**Table 2:** The mean annual employment months, unemployment months, and months spent on disability benefits per different register-based proxies used

Proxies		Total sample		
		Employment	Unemployment	Work disability
Age group	(% of total sample)			
	20–29 (21.1)	8.3	1.1	0.4
	30–39 (23.1)	9.6	1.0	0.5
	40–49 (21.7)	10.1	1.0	0.6
	50–59 (22.8)	9.7	1.1	1.0
	60–64 (11.5)	7.5	1.4	1.8
Sex				
	Male (50.8)	9.1	1.2	0.7
	Female (49.2)	9.4	1.0	0.8
Native language				
	Finnish (86.1)	9.4	1.0	0.8
	Swedish (4.7)	10.0	0.6	0.6
	Other (9.3)	7.3	1.8	0.4
Level of education				
	Primary degree or unknown (13.2)	5.9	2.1	1.8
	Secondary degree (48.0)	9.1	1.2	0.8
	Higher degree (38.8)	10.5	0.6	0.4
Occupational group				
	Armed forces (0.3)	11.8	0.0	0.1
	Managers (2.8)	11.9	0.1	0.1
	Professionals (17.0)	11.7	0.2	0.2
	Technicians and associate professionals (13.7)	11.7	0.2	0.3
	Clerical support workers (3.8)	11.6	0.4	0.3
	Service and sales workers (14.6)	11.5	0.5	0.4
	Skilled agricultural, forestry and fishery workers (1.6)	11.8	0.2	0.4
	Craft and related traders workers (7.3)	11.6	0.4	0.2
	Plant and machine operators, and assemblers (6.2)	11.6	0.4	0.3
	Elementary occupations (4.4)	11.3	0.7	0.4
	Missing information (28.5)	3.3	3.0	2.0
Children under 18				
	0 (68.0)	8.8	1.2	1.0
	1 (13.4)	10.1	0.8	0.4
	2–3 (17.0)	10.5	0.7	0.3
	4 or more (1.6)	9.0	1.0	0.3
Household size				
	1 (25.4)	8.2	1.6	1.2
	2 (33.2)	9.5	1.0	0.7
	3 or more (41.4)	9.7	0.8	0.5
Family type				
	Living alone (25.4)	8.2	1.6	1.2
	Couple without children (28.0)	9.8	0.9	0.7
	Couple with children (34.3)	10.2	0.7	0.4
	Single parent (6.9)	8.6	1.4	0.8
	Missing information (5.4)			
Type of municipality of residence				
	City (75.0)	9.2	1.1	0.7
	Densely populated area (14.0)	9.5	0.9	0.9
	Rural area (11.1)	9.4	1.0	1.0
Number of health challenges				
	0 (50.3)	9.6	1.0	0.3
	1 (39.0)	9.2	1.1	0.8
	2 (10.7)	7.7	1.2	2.5

Two proxies, the level of education and the number of health challenges, show notable variation in work participation status. People with higher education have fewer health challenges and more employment months and fewer unemployment and disability months. Additionally, work participation, measured as months in employment, improves as individuals progress from younger age groups up to the 40–49 age group, where work participation peaks. However, in older age groups, the number of employment months declines. Furthermore, the number of disability months increases with age.

Women have more employment months but also spend more time on disability benefits than men. Swedish speakers have better work participation than Finnish speakers or those speaking other languages. People in densely populated and rural areas have more employment months and fewer unemployment months compared to those living in cities. Additionally, those living with a partner or family generally have higher number of employment months than singles and single parents.

Each occupational group exhibits relatively high employment levels, which is attributable to the construction of data. Specifically, only individuals employed during the last week of the year have an occupation code in the register data. Consequently, those with ‘missing’ codes are often unemployed during this period.

It is worth noting that in most categories, the total number of months spent on various work participation statuses does not equal 12 months. If the sum is less than 12 months, the remaining months are typically spent receiving various allowances and benefits or as a student. Conversely, if the total exceeds 12 months, it may be because a person is employed or unemployed while also receiving a disability benefit.

### Work Ability Clusters

Using the same register-based proxies for factors and determinants of work ability, the working-age population in Finland was next organized into 11 clusters. Clusters were created to illustrate the combined effect of the variables examined in the previous chapter on the working-age population, and to analyze how these variables and their values intersect within the population. The names and descriptions of the work ability clusters are provided in Table 3. The descriptions of the work ability clusters are indicative and are based on the more detailed data provided in Table 4 in appendix which, in addition to presenting the mean age for the total sample and various clusters, provides the relative frequencies of work ability-related register-based proxies for both the entire sample of the working-age population and the work ability clusters. The information provided can be used to distinguish work ability clusters from one another as well as from the total sample of the working-age population. The reasoning behind the wording used in the descriptions is provided in Table 5 in Appendix.

**Table 3:** Descriptions of work ability clusters

Cluster title	Description
Cluster 1: Young people with intermediate to high education living alone in a city, good health	Includes young people (mean age 28 years), most of whom live alone and in a city. The cluster includes more people who live alone than any other cluster (88%). Additionally, 99.6% of them have no underaged children. Most have a secondary education degree, while 19% hold a higher education degree. More than two-thirds are men. They have little health challenges.
Cluster 2: Young women with low to intermediate education living in a relationship in a city, intermediate health	Includes young women (mean age 29 years) who live in a city. They typically have a low level of education, and over 60% of them are in a relationship without children. Additionally, more than 90% of them live together with another adult. They have some health challenges.
Cluster 3: Young men with low to intermediate education, living in a relationship in a city, good health	Includes young men (mean age 32 years) who live in a city. They typically have a low level of education, and over 60% of them are in a relationship without children. More than 90% of them live with another adult. The cluster includes more people working in construction, repair, manufacturing, process, and transport workers compared to other clusters. They have little health challenges.
Cluster 4: Middle-aged people with low to intermediate education living with a family, intermediate health	Includes middle-aged individuals (mean age 37 years), most of whom have a secondary degree education. The vast majority (88.9%) live in families with at least one child. This cluster typically has larger households, usually consisting of 3 to 5 people. Two-thirds of the individuals in this cluster are women. Over 14% speak a language other than Finnish or Swedish as their native language. They have some health challenges.
Cluster 5: Middle-aged people with high education living in a relationship in a city, good health	Includes middle-aged individuals (mean age 39 years), most of whom are in relationships without children (77%). Over 85% of them live in a city. The majority are women (83%), and nearly all (96%) have a higher degree education. More than half of the individuals in this cluster are professionals. They have little health challenges.
Cluster 6: Middle-aged women with high education living with a family, good health	Includes middle-aged women (mean age 43 years) who typically have a high level of education. Almost all of them (95%) live in families with children. This cluster has a higher proportion of technicians and associate professionals compared to other clusters. They have little health challenges.
Cluster 7: Middle-aged men with intermediate to high education living with a family, intermediate health	Includes middle-aged men (mean age 43 years), 60% of whom have a higher degree education. The vast majority live with another adult and at least one child. This cluster also has a higher proportion of leaders and more people who speak Swedish as their native language compared to other clusters. They have some health challenges.
Cluster 8: Over middle-aged people with high education, single parent households, poor health	Includes over middle-aged people (mean age 53 years), over 80% of whom have a high level of education. Almost all (96%) are women, and more than one-third are single parents. They have health challenges.
Cluster 9: Over middle-aged people with intermediate to high education living alone, poor health	Includes over middle-aged people (mean age 55 years old), most of whom (87%) live alone. About two-thirds of them are men. People in this cluster typically have a low level of education. The cluster also includes more people who speak Finnish as their native language than any other cluster. They have health challenges.
Cluster 10: Over middle-aged people with low to intermediate education living in a relationship in rural or densely populated areas, poor health	Includes over middle-aged people (mean age 56 years old), most of whom are in relationships and live with another adult. Over 40% of them live in rural areas, while about one-fourth reside in densely populated areas. The majority are women, and the cluster contains a higher proportion of farmers and people who speak Swedish as their native language compared to other clusters. They have health challenges.
Cluster 11: Over middle-aged people with intermediate to high education living in a relationship, poor health	Includes primarily over middle-aged people (mean age 59 years), who live in a relationship with no children aged under 18 living in the same household. Over 70% of them are men, and nearly half have a higher degree education. They have health challenges.

### Work Participation Statuses Across the Work Ability Clusters

**Fig. 1:**
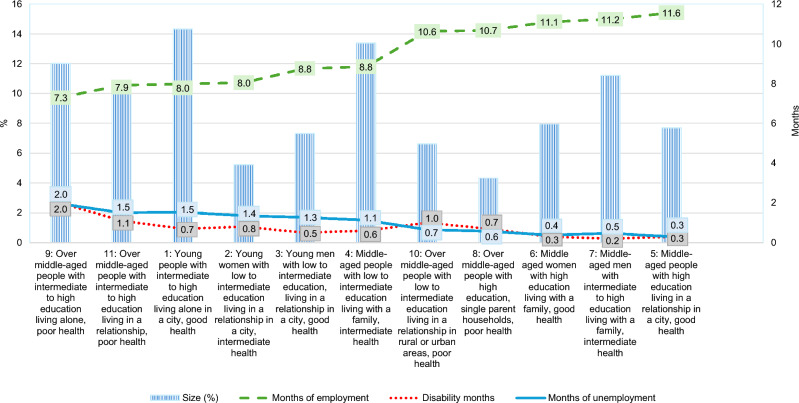
Work participation statuses in the work ability clusters

The work participation statuses of the clusters are portrayed in Fig. 1. The sizes of the clusters are represented as bars in the chart and range between 5.2% (Cluster 2, “Young women with low to intermediate education living in a relationship in a city, intermediate health”) and 14.3% (Cluster 1, “Young people with intermediate to high education living alone in a city, good health”) of the total sample. The average number of months employed in the clusters ranged from 7.3 in Cluster 9 (“Over middle-aged people with intermediate to high education living alone, poor health”) to 11.6 in Cluster 5 (“Middle-aged people with high education living in a relationship in a city, good health”). In terms of unemployment months, Cluster 5 had the lowest average at 0.3 months, while Cluster 9 showed the highest average at 2.0 months. Additionally, disability months averaged from 0.2 months in Cluster 7 (“Middle-aged men with intermediate to high education living with a family, intermediate health”) to 2 in Cluster 9.

From Fig. 1, it can be observed that, in contrast to the ascending dashed line representing employment months, the solid line for unemployment months is descending: on average, clusters with more employment months have fewer unemployment months and vice versa. In total, the months spent on different work participation statuses approach 12 months, so reduced employment in a cluster is primarily reflected in unemployment and disability months. Disability months are not directly inversely proportional to employment months like unemployment months are instead, in a few clusters, they are high despite relatively high work participation. Additionally, clusters 8, “Over middle-aged people with high education, single parent households, poor health” and 10 “Over middle-aged people with low to intermediate education living in a relationship in rural or densely populated areas, poor health” are the only ones where disability months exceed unemployment months.

The distribution of employment months, unemployment months, and months spent on disability benefits for each cluster is detailed in Appendix Tables 6, 7, and 8, 9, respectively. For nearly all clusters, the distributions of employment and unemployment months exhibit a U-shaped pattern: the most common durations are either 0 or 12 months. In terms of months spent on disability benefits, some clusters show a tendency towards shorter durations, while 12 months is also a frequent occurrence. This pattern indicates that the reported averages are based on these U-shaped distributions.

### Sensitivity Analysis

As the work ability clustering result fully depends on the variables used, we conducted a sensitivity analysis that excluded the measure for health challenges. This analysis was performed to evaluate the robustness of our findings and to determine the impact of including or excluding health challenges on the identified clusters. Essentially, this involved conducting the clustering using other work ability proxies besides health challenges, providing insight into whether the cluster results depend on including health challenges. As the number and composition of clusters varied significantly when health challenges proxy was left out, we concluded that its inclusion, despite consisting of only two variables (the purchase of medications under the special reimbursement system of the Social Insurance Institution and the use of specialized health care), is justified as part of the work ability construction. Sensitivity analysis resulted in six distinct clusters, which are described in Table 10 in the Appendix.

## Discussion

In this cross-sectional exploratory study, we operationalized the multifaceted and complex concept of work ability in individual level using register data from the Finnish working-age population. We clustered the population based on register-based proxies related to work ability. A total number of 11 clusters were formed. These work ability clusters were then compared based on their characteristics, as well as their average number of annual employment months, unemployment months, and months spent on disability benefits. Whereas clustering analysis has been used in the context of work ability and work participation previously to identify employment barriers by clustering unemployed individuals using survey-based data [21], to the best of our knowledge, this is the first study to operationalize and examine work ability and work participation using register data of working-age population.

In their cross-disciplinary synthesis of the work ability literature, Cadiz et al. [11] highlight a few potential pathways for future research: avoiding the use of single measures for work ability and employing work ability-related factors and outcomes. Partly adapting this approach, we identified clusters based on factors related to work ability on individual level and propose ways to leverage this information to enhance workforce participation. Our operationalization of the work ability construct, informed by Lederer et al. [10], considers its multidimensional nature. Unlike traditional assessments of work ability that use survey data and a normative scale, or measure of work disability, our approach and methodology focus on describing the accumulation of factors related to work ability within individuals, which we call collective contribution.

The number of sickness allowance days, a known measure of work disability and a potential indicator of health challenges available in the data was not included in our main analysis as a variable used in clustering analysis. This measure could introduce bias in our analysis, as it would function both as a clustering variable and as an outcome measure for the number of months receiving work disability benefits. To emphasize ability rather than absence and to avoid potential systematic bias, we excluded this measure from the list of health challenges used in the analysis. Shifting the perspective from individual work ability measures, work disability measures, and their associations with work participation to a broader view of collective contribution to work ability revealed a nuanced relationship between demographic characteristics, health challenges, work ability, and work participation. As an example, while examining health challenges from a single measure perspective showed a negative correlation with employment months, the cluster analysis indicated that work ability clusters with more health challenges did not consistently result in fewer months of employment. Several explanations could account for this: some health challenges may not significantly impact functional capacity and, thus, work ability, others can be effectively managed through medication, treatment, and work ability management, and individuals may continue working despite minor health challenges to fulfill financial requirements. However, it is likely that other factors or determinants of work ability besides health play a role in work participation among those with health issues. For example, Clusters 9 and 11 with below-average employment months, poor health may be dominant factor despite higher education levels. For Clusters 2 and 4, they exhibit intermediate health, suggesting that while health may not be an immediate barrier, when combined with low education, may show as lower employment. Conversely, Clusters 1 and 3 with good health, the low to intermediate education level in Cluster 3 and, in the case of Cluster 1, the prevalence of living alone, appear to offset the benefits of good health.

Regarding factors or determinants potentially related to work ability, there has been progress in the literature on working life expectancy. Burdorf et al. [13] list various barriers or facilitators for working life expectancy, and Solovieva et al. [14] reviewed literature on socioeconomic differences in working life expectancy. While the working life expectancy literature increasingly focuses on barriers or facilitators of work participation—including potential determinants of work ability—it remains primarily centered on individual factors instead of collective contribution or cumulative effect. This progress, however, points to directions for future studies on work ability and work participation, advocating for analyses that extend over longer time spans instead of relying solely on cross-sectional views.

## Using Work Ability Cluster Information to Inform Policymakers

Identifying specific groups based on work ability and related factors can aid in targeting interventions or support measures aimed at improving work participation, healthcare provision, and social services, or reduction of social inequity for these groups. Clustering analysis plays a crucial role in pinpointing specific groups within the working-age population that can benefit from targeted interventions.

Our example focuses on what is termed workforce engagement potential, indicated by an underutilization of capacity amounting to fewer than 12 annual months of employment. Such potential can be identified, for instance, in Cluster 11, which shows an average of 7.9 months of employment, 1.5 months of unemployment, and 1.1 months spent on disability benefits. There are several reasons and determinants for work participation that our analysis did not consider, and so the results should be read in the scope of work ability-related issues. Moreover, our analysis did not have data from workplaces and work arrangements. However, the data that we had about various themes of work ability according to Lederer et al. framework, included 90% of the working-age people in Finland and thus leaves little room for speculation about the results as we presented them. With the limitations in mind, the following notions and suggestions can be made.

Cluster 11 predominantly comprises individuals from older age groups and is characterized by poor health. To achieve full or near-full workforce engagement potential for this specific cluster, policies could focus on reducing unemployment and supporting work ability tailored to the needs of older individuals. For example, for the unemployed, managing health and disabilities and creating opportunities for employment could further enhance their participation in the workforce [22]. For the employed, it is also important to address health, as longitudinal evidence from Finland suggests a health-based selection effect leading to unemployment among those with relatively poor health [23]. Workplace accommodations such as vocational counseling and guidance, work organization, and changes of work schedules could be implemented to enhance the work participation of individuals experiencing challenges related to health and work disability [24]. Moreover, targeted policy interventions to support the active participation of older workers in the labor market include providing training and skill development through ongoing learning opportunities and retraining options [1]. The average age in Cluster 11 is higher than in any other cluster, and its work participation is lower compared to clusters primarily composed of middle-aged individuals, indicating an age-related disparity. Combating age discrimination is key to promoting social equity among older age clusters, which can be achieved through education on the value of age diversity and enforcing anti-discrimination policies [1].

### Strengths

The study’s main strength lies in its utilization of comprehensive and detailed objective administrative data, capturing a 90% sample of the total working-age population in Finland. This extensive dataset significantly reduces potential biases often associated with self-reported data. Additionally, the richness of the data, which includes numerous register-based proxies on factors and determinants related to work ability, provides a robust foundation for thorough analysis and thus serves as a foundation for our aim to operationalize work ability using register data. A methodological novelty in the study is the use of K-prototypes clustering in the context of work ability research, which allows for the inclusion of both continuous and categorical variables. This approach enhances the capacity to uncover complex relationships within the data, offering insights into the proxies of work ability. Using register data, it is possible to monitor the situation without laborious population sample studies.

The obvious strength of the study is its criterion validity, as we used register data. Thus, if the work ability construct is deemed reasonable as we present it, it can be predicted using the selected indicators. Content validity is at a fairly good level because various work ability-related variables are used from different dimensions, although the lack of workplace data is a clear limitation. Construct validity cannot be assessed using existing principles since we introduced a new construct of work ability. Future research will reveal how our results align with self-evaluation outcomes regarding work ability or other relevant comparisons.

### Limitations

This study has some limitations. First, the register data primarily focus on one level of work ability—the individual level—as discussed by Lederer et al. [10]. Consequently, this study explores individual-level register-based proxies work ability and is unable to focus on the organizational and societal levels. As a result, important dimensions such as physical, mental, emotional, and social aspects of work, along with organizational culture and access to occupational healthcare services, may not be fully captured, thus limiting the perspective on determinants of work ability. However, incorporating occupational information can partially address the organizational level, since different dimensions of work play varying roles across occupations. Future research should aim to more comprehensively examine the organizational level. Second, there is missing information regarding some register-based proxies for work ability factors or determinants, such as the level of education and occupations. We categorized missing information, such as individuals who were unemployed in the last week of 2021 and thus lacked occupation data, by placing them into a “missing” category. Clustering based on “missing” category may have introduced bias to our results. For example, “missing” category may have potentially clustered all unemployed individuals in the same group. Another example of “missing” category with potential bias involves individuals who obtained their education abroad, as these educational degrees are often not recognized in Finnish registers. Third, the k-prototypes clustering method is not without biases. One source of bias is the choice of the number of clusters, which could lead to underfitting or overfitting. Additionally, there can be issues with initialization, such as poor placement of cluster centroids. In the current study, the number of clusters was determined based on distance measures and qualitative evaluation of the clusters. We observed no signs of overfitting, such as a small number of individuals in clusters or high similarity between clusters. Regarding underfitting or initialization issues, we provided evidence of the differences between clusters and the similarity of individuals within clusters. Fourth, the data are limited to the Finnish working-age population, raising questions about the generalizability and external validity of these findings. Based on the current analysis, it cannot be confirmed that the results are applicable to countries other than Finland. Generalizability should be assessed by conducting similar analyses with data from other countries using comparable operationalizations of the proxies. Fifth, regarding family type, it should be noted that individuals over 18 living with their parents are categorized according to their parents’ family type. For example, someone classified as “couple with children” may not be part of the couple but rather a child over 18 living with them. A final limitation of the study’s data is the absence of observations from individuals who passed away during 2021. This gap, due to the dataset’s construction, could result in an incomplete representation of the population, potentially skewing findings, particularly concerning those with serious health issues. Consequently, if the work ability-related factors of those who died in 2021 were distinct, the analysis would not capture these associations or related insights. However, the individuals from the working-age population who died in 2021 represent less than half a percent of the overall working-age population.

Additionally, the analysis also has its limitations. It is important to note that while we describe clusters based on average characteristics, not all individuals in a cluster fully represent the average profile. Second, most proxies were operationalized as categorical variables. Altering categories or representing them as continuous variables—like using the number of children as a continuous variable—could influence the clustering outcomes. Third, although there are administrative register proxies on work disability, we did not include them in our clustering analysis to focus more on work ability proxies. Including them might have identified clusters using work disability benefits. We utilized months in disability benefits as an outcome measure. Fourth, we employed very crude measures of health, limiting health proxies to two health challenges. Future studies should consider distinguishing between physical and mental health in individual-level proxies.

Finally, this is an exploratory study aiming to operationalize work ability. As reviewed by Lederer et al. [10], work ability is a highly multifaceted and complex concept, and the question remains whether it can be comprehensively operationalized at different levels. This study represents a significant advancement in operationalizing work ability from register data and analyzing the collective contribution of these register-based proxies on factors and determinants related to work ability.

## Conclusions

This study translated the complex concept of individual-level work ability into measurable terms using extensive register data, leading to the identification of 11 distinct clusters within the Finnish working-age population. The observed variations among these work ability clusters—in terms of age, sex, educational attainment, occupation, household size, family type, and health status—highlight the intricate and diverse nature of work ability. Importantly, the differing work participation statuses across these work ability clusters illustrate the subtle interactions of these variables in shaping employment, unemployment, and dependency on disability benefits.

In assessing and enhancing the work ability of the working-age population, it is crucial to consider the dynamic interactions and potential accumulation of factors contributing to work ability, referred to here as the collective contribution, rather than focusing on individual factors alone. Identifying these interactions may aid in tailoring interventions and policies to the unique characteristics and needs of each cluster. Furthermore, this study demonstrates the utility of register data in capturing and analyzing work ability, offering a valuable framework for future research and policy development.

## Data Availability

The results of our study are based on the “State of Work Ability in Finland” register data generated by the Finnish Institute of Occupational Health. The register data consist on data provided by Statistics Finland, the Finnish Institute for Health and Welfare, the Finnish Centre for Pensions, and Kela, the Social Insurance Institution of Finland. Restrictions apply to the availability of these data, which were used under license in a remote access environment for the current study and are not publicly available. The use of the data requires permissions that are available for a fee. Therefore, the data cannot be shared.

## Appendix

See Tables 4, 5, 6, 7, 8, 9, and 10.

**Table 4:** The mean or relative frequencies of the register proxies in the total sample and individual clusters

Proxies		Total sample	Cluster 1	Cluster 2	Cluster 3	Cluster 4	Cluster 5	Cluster 6	Cluster 7	Cluster 8	Cluster 9	Cluster 10	Cluster 11
Number of individuals, thousands		2750.7	394	143.6	201	368.4	211.9	218.8	308	119.2	330	182.2	273.7
Proportion of total sample, %		100	14.3	5.2	7.3	13.4	7.7	8	11.2	4.3	12	6.6	10
Age, mean		42.4	28.1	28.9	31.7	36.5	39.4	42.5	43.3	52.6	54.6	56	58.6
Age class, %													
	20–29	20.9	63.9	62.3	43.5	18.1	23.1	6.7	4.4	0.1	0	0	0
	30–39	23.1	31.3	26.7	36	50.2	34.3	25.6	24	3.7	0	1	0
	40–49	21.7	4.8	10	16.7	25.7	19	42.3	56.8	24.4	23.6	11.3	4.8
	50–59	22.8	0	1.1	0.8	5.9	19.5	23.1	14.7	57.5	48.7	58.6	44.4
	60–64	11.5	0	0	0	0.2	4.1	2.3	0.2	14.3	27.7	29.1	50.8
Sex, %													
	Male	50.8	68	0	100	34.6	17	0	100	4.3	65.6	18.5	74.3
	Female	49.2	32	100	0	65.4	83	100	0	95.7	34.4	81.5	25.7
Native language, %													
	Finnish	86.1	87.5	83.7	82.2	80.9	87.9	86.4	83.4	86.1	89.8	89.6	89.4
	Swedish	4.7	4.2	3.6	4.2	4.8	4.8	5.6	5.8	5.2	3.7	6.1	4.3
	Other	9.3	8.3	12.7	13.6	14.4	7.3	8.1	10.8	8.7	6.6	4.3	6.4
Level of education, %													
	Primary degree or unknown	13.2	16.1	14.6	18.7	12.8	2.9	4.2	13.1	10.1	18.2	8	19.4
	Secondary degree	48	64.8	77.4	68	73.7	1	11.3	26.6	8.7	57.2	83.1	31.5
	Higher degree	38.8	19.1	8.1	13.3	13.5	96.1	84.5	60.3	81.2	24.7	9	49.1
Occupation, %													
	Armed forces	0.3	0.3	0	0.6	0.2	0.1	0	1	0.1	0.2	0	0.2
	Managers	2.8	0.6	0.3	1.5	2	2.2	3.5	7.8	3.6	2	1.4	5.2
	Professionals	17	7.6	2	3.6	1.6	56.6	42.4	33.6	38.9	6.9	2.8	11.1
	Technicians and associate professionals	13.7	8.7	5.5	11	11.8	23.2	23.6	16.2	22.1	9.9	9.8	14.6
	Clerical support workers	3.8	3.6	4.9	3	3.5	5.5	5	1.9	6.2	3.3	5.1	3.1
	Service and sales workers	14.6	14.6	28.8	11.2	22.6	6.2	12.2	7	8.5	10.3	43.5	4
	Skilled agricultural, forestry, and fishery workers	1.6	0.8	0.7	1.3	1.8	0.4	1.1	2.4	1.3	1.7	4	1.7
	Craft and related traders workers	7.3	8.5	2	16	9.4	0.6	0.8	10.9	1.4	8.3	5.5	8.3
	Plant and machine operators and assemblers	6.2	7.2	3	11.2	7.1	1.1	1.4	8.4	1.9	7.5	6.5	6.7
	Elementary occupations	4.4	5.6	6.7	5.6	5	1.8	2.7	2.8	3.9	4.7	8.5	2.2
	Missing information	28.5	34.9	46.1	35.1	45.3	2.4	8	7.4	12.1	42.7	12.9	42.8
Number of underaged children, %													
	0	68	99.6	87.2	97.9	9.4	97.3	12.9	2.7	78.3	99.5	96.2	99.3
	1	13.4	0.41	12.8	2.1	12.6	2.8	48.6	56	8.4	0.4	3.5	0.6
	2–3	17	0	0	0	71.8	0	34	37.9	12.5	0.1	0.2	0.1
	4	1.6	0	0	0	6.2	0	4.5	3.4	0.8	0	0.1	0.01
Household size, %													
	1	25.9	89.6	0	0	0	14.3	0	0	24	89.3	4.8	0
	2	33.8	0	90.8	90.2	0	83.3	3	0.9	15.3	1.6	84.1	94.4
	3 or more	40.3	10.4	9.2	9.8	100	2.4	97	99.1	60.8	9.1	11.1	5.6
Family type, %													
	Living alone	25.4	87.7	0	0	0	14.3	0	0	22.8	86.9	4.8	0
	Couple without children	28	0	63.5	66	0	77	0	0	3	0.3	76.1	87.8
	Couple with child(ren)	34.3	6.8	0	1.9	88.9	0	95.2	96.1	30.4	7	7.7	2.4
	Single parent	6.9	1.4	18.8	11.1	9.8	3.9	4.8	2.2	35.2	1.5	8.2	4.5
Type of municipality of residence, %													
	City	74.9	84.7	84.6	80.9	68	86.7	75.7	74.7	79	74.8	34.1	77
	Densely populated area	14	9.1	9.6	11.3	18.1	9.3	14.6	14.8	12.9	14.6	23.9	15.1
	Rural area	11.1	6.2	5.8	7.8	13.9	4	9.7	10.5	8.1	10.6	42	7.9
Number of health challenges, %													
	0 health challenges	50.3	56.9	48.6	63.3	49.6	50.6	61.1	45.2	43.9	43.2	41.9	41.6
	1 health challenge	39	36.5	43.9	31.3	43.3	40.8	32.1	46.1	42.4	37.8	41.4	39
	2 health challenges	10.7	6.5	7.5	5.5	7.1	8.6	6.9	8.7	13.7	19	16.7	19.5

The choice of the number of clusters was based on calculating the sum of the distances of all points to their respective cluster centroids. Minimizing this distance measure indicates that individuals, on average, are closer to the cluster centroids. Table 5 presents the number of clusters and the associated distance measures. Local minima are highlighted in bold.

**Table 5:** Number of clusters and associated distance measures

Number of clusters	Distance measure	Number of clusters	Distance measure
2	1,818,480	**11**	**1,109,179**
3	1,644,881	12	1,115,380
4	1,457,897	13	1,085,665
5	1,408,435	14	1,049,739
6	1,327,561	**15**	**1,034,127**
7	1,277,771	16	1,042,645
8	1,217,703	**17**	**1,008,666**
9	1,171,456	18	1,079,886
10	1,144,352	19	1,009,800
		**20**	**1,004,439**

Table 6 provides background information that forms the basis for the reasoning behind the cluster descriptions.

**Table 6:** The reasoning behind the cluster descriptions

Age			Clusters
	Young	Mean age of the cluster under 35	1, 2, 3
	Middle-aged	Mean age of the cluster 35–50	4, 5, 6, 7
	Over middle-aged	Mean age of the cluster over 50	8, 9, 10, 11
Level of education			
	Low or intermediate	Under 15% of the individuals in the cluster have a higher degree and more individuals have a primary or unknown degree than a higher degree	2, 3, 4, 10
	Intermediate to high	Over 15% of the individuals in the cluster have a higher degree and more individuals have a secondary degree than primary or unknown degree	1, 7, 9, 11
	High	Over 80% of the individuals in the cluster have a higher degree	5, 6, 8
Family type			
	Living alone	Over 80% of the individuals in the cluster live alone	1, 9
	Living in a relationship	Over 60% of the individuals in the cluster live with another adult	2, 3, 5, 10, 11
	Living with a family	Over 80% of the individuals in the cluster live in with another adult and at least one child	4, 6, 7
	Single parent	Over 30% of the individuals in the cluster live with at least one child	8
Sex			
	Male	100% of the people in the cluster are men	3, 7
	Female	100% of the people in the cluster are women	2, 6
Health			
	Good	Over 50% of the people in the cluster have no health challenges	1, 3, 5, 6
	Intermediate	Over 40% of the individuals in the cluster have one health challenge	2, 4, 7
	Poor	Over 10% of the individuals in the cluster have two health challenges and less than 50% of them have no health challenges	8, 9, 10, 11
Type of municipality of residence			
	City	Over 80% of the individuals in the cluster live in a city	1, 2, 3, 5
	Urban or rural	Over 60% of the individuals in the cluster live either in an urban or rural area	10

**Table 7:** Distribution of employment months in clusters

Employment months												
	Cluster 1	Cluster 2	Cluster 3	Cluster 4	Cluster 5	Cluster 6	Cluster 7	Cluster 8	Cluster 9	Cluster 10	Cluster 11	Total
0	22.4	22.0	18.7	18.7	1.2	3.4	4.0	8.7	34.3	8.6	28.1	16.9
1	1.6	1.6	1.1	1.1	0.2	0.3	0.3	0.3	0.9	0.4	1.0	0.9
2	1.6	1.5	1.2	1.1	0.3	0.4	0.4	0.3	0.9	0.4	0.9	0.9
3	1.7	1.5	1.2	0.9	0.3	0.4	0.3	0.3	0.7	0.4	0.9	0.8
4	2.2	1.9	1.6	1.2	0.4	0.5	0.4	0.4	1.0	0.5	1.1	1.1
5	1.7	1.7	1.3	1.2	0.4	0.6	0.4	0.4	0.7	0.5	0.9	0.9
6	2.2	2.2	1.7	1.9	0.5	1.1	0.5	0.5	1.1	0.7	1.4	1.3
7	2.2	2.3	1.7	1.8	0.7	1.3	0.5	0.6	0.9	0.6	1.1	1.3
8	3.8	3.8	2.8	2.2	0.9	1.4	0.7	0.7	1.1	0.8	1.3	1.8
9	2.8	3.1	2.3	2.0	0.9	1.2	0.8	0.7	1.1	0.8	1.4	1.6
10	2.4	2.8	2.1	2.0	1.1	1.3	0.9	0.7	1.2	0.9	1.3	1.6
11	3.0	3.6	2.9	2.7	1.7	1.8	1.4	1.0	1.6	1.3	1.8	2.1
12	52.5	52.0	61.5	63.1	91.6	86.5	89.4	85.4	54.6	84.3	58.9	68.8

**Table 8:** Distribution of unemployment months in clusters

Unemployment months												
	Cluster 1	Cluster 2	Cluster 3	Cluster 4	Cluster 5	Cluster 6	Cluster 7	Cluster 8	Cluster 9	Cluster 10	Cluster 11	Total
0	75.3	76.2	79.0	80.0	92.3	91.6	91.1	90.5	75.7	88.1	81.3	82.8
1	3.3	3.7	3.1	3.1	1.8	1.7	1.6	1.3	1.9	2.1	1.4	2.3
2	2.8	3.2	2.5	2.9	1.9	1.8	1.3	1.3	1.6	1.8	1.2	2.1
3	2.2	2.4	2.0	2.1	1.0	1.0	1.0	0.9	1.4	1.1	1.2	1.5
4	2.1	2.0	1.8	1.7	0.7	0.7	0.9	0.8	1.4	0.9	1.1	1.3
5	1.8	1.8	1.6	1.4	0.6	0.6	0.7	0.7	1.3	0.9	1.1	1.2
6	1.6	1.4	1.3	1.2	0.4	0.4	0.5	0.6	1.4	0.7	1.1	1.0
7	1.5	1.5	1.2	1.2	0.4	0.5	0.5	0.5	1.2	0.6	0.9	1.0
8	1.6	1.4	1.2	1.1	0.3	0.4	0.4	0.5	1.2	0.6	0.9	0.9
9	1.2	1.1	1.0	0.9	0.2	0.3	0.4	0.4	1.3	0.5	0.9	0.8
10	1.1	0.8	0.9	0.7	0.2	0.2	0.3	0.4	1.0	0.4	0.7	0.7
11	1.1	0.9	0.8	0.7	0.1	0.2	0.3	0.3	1.1	0.4	0.7	0.6
12	4.5	3.6	3.8	3.2	0.3	0.7	1.2	1.9	9.4	1.8	7.5	3.8

**Table 9:** Distribution of disability months in clusters

Disability months												
	Cluster 1	Cluster 2	Cluster 3	Cluster 4	Cluster 5	Cluster 6	Cluster 7	Cluster 8	Cluster 9	Cluster 10	Cluster 11	Total
0	89.1	86.6	91.3	87.7	85.2	89.5	93.6	86.6	76.2	80.0	83.5	86.7
1	2.2	3.2	2.0	3.7	4.5	4.3	2.4	3.2	3.0	5.4	2.7	3.2
2	1.7	2.3	1.5	2.6	3.0	2.7	1.5	2.3	2.5	3.9	2.4	2.3
3	0.9	1.2	0.7	1.2	1.4	1.1	0.7	1.2	1.2	1.9	1.1	1.1
4	0.6	0.8	0.4	0.7	0.9	0.6	0.4	0.8	0.9	1.3	1.0	0.7
5	0.4	0.5	0.3	0.4	0.5	0.4	0.2	0.5	0.6	0.9	0.6	0.5
6	0.3	0.4	0.2	0.3	0,4	0.3	0.2	0.4	0.5	0.6	0.5	0.4
7	0.4	0.5	0.2	0.4	0.5	0.3	0.2	0.5	0.7	0.9	0.5	0.4
8	0.2	0.3	0.1	0.2	0.3	0.2	0.1	0.3	0.4	0.4	0.4	0.3
9	0.2	0.3	0.1	0.2	0.2	0.1	0.1	0.3	0.3	0.4	0.4	0.2
10	0.2	0.2	0.1	0.2	0.2	0.1	0.1	0.2	0.3	0.3	0.3	0.2
11	0.2	0.2	0.1	0.1	0.1	0.1	0.1	0.2	0.3	0.3	0.3	0.2
12	3.7	3.6	3.0	2.3	2.9	0.5	0.6	3.6	13.2	3.9	6.4	4.0

**Table 10:** Sensitivity analysis: Results

Clusters	Descriptions
Cluster 1	Includes young people (mean age 27 years), most of whom live alone in a city. Most of them have a secondary degree and 24% of them have a higher education. Most of them are women. They have little health challenges (good health)
Cluster 2	Includes middle-aged people (mean age 39 years), most of whom live in a relationship and 10% of them are single parents. Most of them have a secondary education and 18% of them have a higher degree education. More than three-fourth of them are men. They have little health challenges (good health)
Cluster 3	Includes over middle-aged people (mean age 54 years), most of whom live in a relationship. Most of them have a higher degree education. 80% of the individuals in this cluster are women. They have health challenges (poor health)
Cluster 4	Includes middle-aged people (mean age 42 years), most of whom live with a family. Most of them have a higher degree education and 70% of them are women. They have some health challenges (intermediate health)
Cluster 5	Includes middle-aged people (mean age 39 years), most of whom live with a family. Most of them have a secondary degree education and over three fourth of them are men. They have little health challenges (good health)
Cluster 6	Includes over middle-aged people (mean age 52 years), most of whom live alone. Most of them have a secondary degree education and 25% have a higher degree education. 70% of the individuals in this cluster are men. They have health challenges (poor health)

Additional information on the health challenge indicators used:

1. Purchase of medications under the special reimbursement system of the Social Insurance Institution.

The Pharmaceuticals Pricing Board (Hila) can grant special reimbursement status to medications used in the treatment of severe and chronic illnesses, such as epilepsy, various cancers, asthma, diabetes, multiple sclerosis, inflammatory bowel disease, chronic coronary heart disease, and rheumatism. These illnesses are defined in a government decree [25].

2. Use of specialized health care.

Specialized healthcare in Finland includes various specialty examinations and treatments primarily offered in hospitals, outpatient clinics, and emergency departments. Access usually requires a referral, except for urgent care. Public hospitals, including regional central hospitals and university hospitals, provide both basic and advanced specialized care, with university hospitals handling the most complex cases. Private hospitals complement public services, funded through patient payments, insurance, or regional acquisitions [26].

## Declarations

### Research Involving Human/Animal Participants

No animal or human studies were carried out by the authors for this article.
